# Severe mycosis as a rare infection after a corn auger injury of the hand: a case report

**DOI:** 10.1186/s13037-015-0061-x

**Published:** 2015-05-24

**Authors:** Richard J Bowles, Justin J Mitchell, Connie Price, Kyros Ipaktchi

**Affiliations:** 1 Department of Orthopaedic Surgery, University of Colorado School of Medicine, 12631 E. 17th Avenue, Aurora, CO 80045 USA; 2 Department of Medicine, Infectious Diseases, Denver Health Medical Center, 777 Bannock Street, Denver, CO 80204 USA; 3 Department of Orthopedic Surgery, Denver Health Medical Center, 777 Bannock St, Denver, CO 80204 USA

**Keywords:** Agricultural injuries, Mangled hand, Mucormycosis, Zygomycosis

## Abstract

Mucormycosis is a rare but serious infection that can be seen in immunocompetent individuals who experience traumatic injury. The authors report a case in a 28 year-old man who sustained a mangling hand injury in a corn augur accident. After initial aggressive debridement ongoing tissue necrosis was seen, and in subsequent biopsies invasive mucormycosis was diagnosed. The patient was successfully managed with immediate surgical debridement and antifungal medication and showed no sign of infection at six-month follow-up.

## Background

Mucormycosis, formerly known as zygomycosis, is a rare infection caused by fungal species that are commonly found in soil and decaying organic matter. Though mucormycosis is more likely in immunocompromised individuals, traumatic injury may allow penetration of the fungus through the mucocutaneous barrier and aggressive infection may result. Typical for this infection is the “angioinvasive” spreading of the disease process which can result in a risk for loss of limb and life if not rapidly controlled. Identification and treatment of this serious infection is important as the mortality rate associated with cutaneous mucormycosis has been shown to be as high as 31% [1].

In this case report we describe an immunocompetent patient involved in an agricultural accident who sustained a mangled left hand that was complicated by a subsequent mycotic infection.

## Case presentation

**Figure 1:**
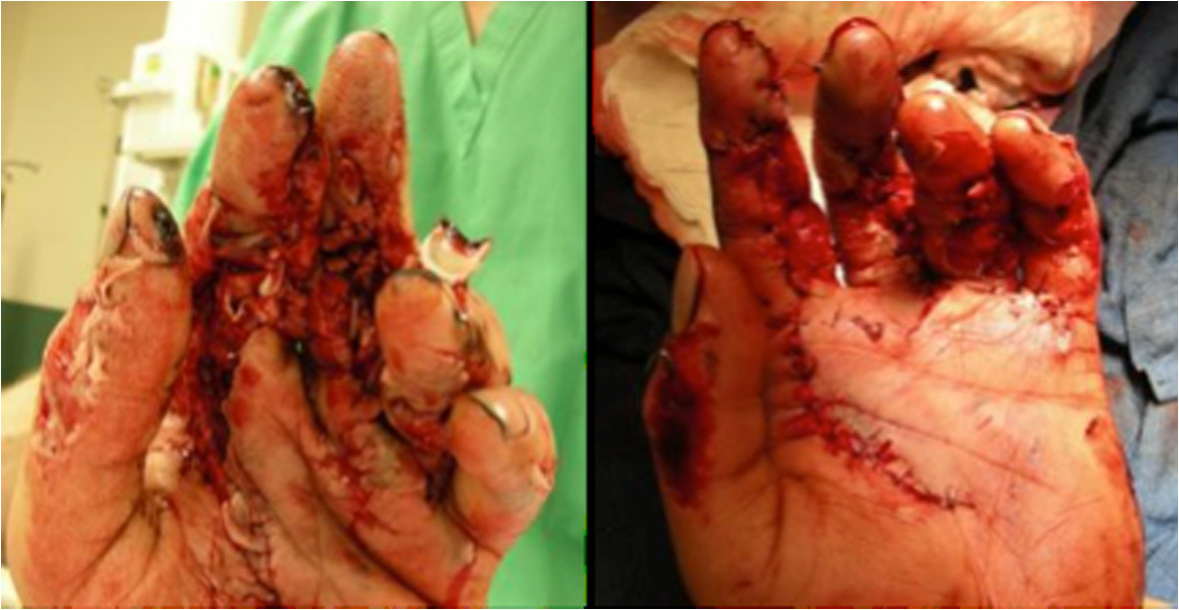
Preoperative situation demonstrating extensive soft tissue laceration by corn grinding machinery (left). Postoperative view after irrigation and debridement with partial soft tissue closure (right).

**Figure 2:**
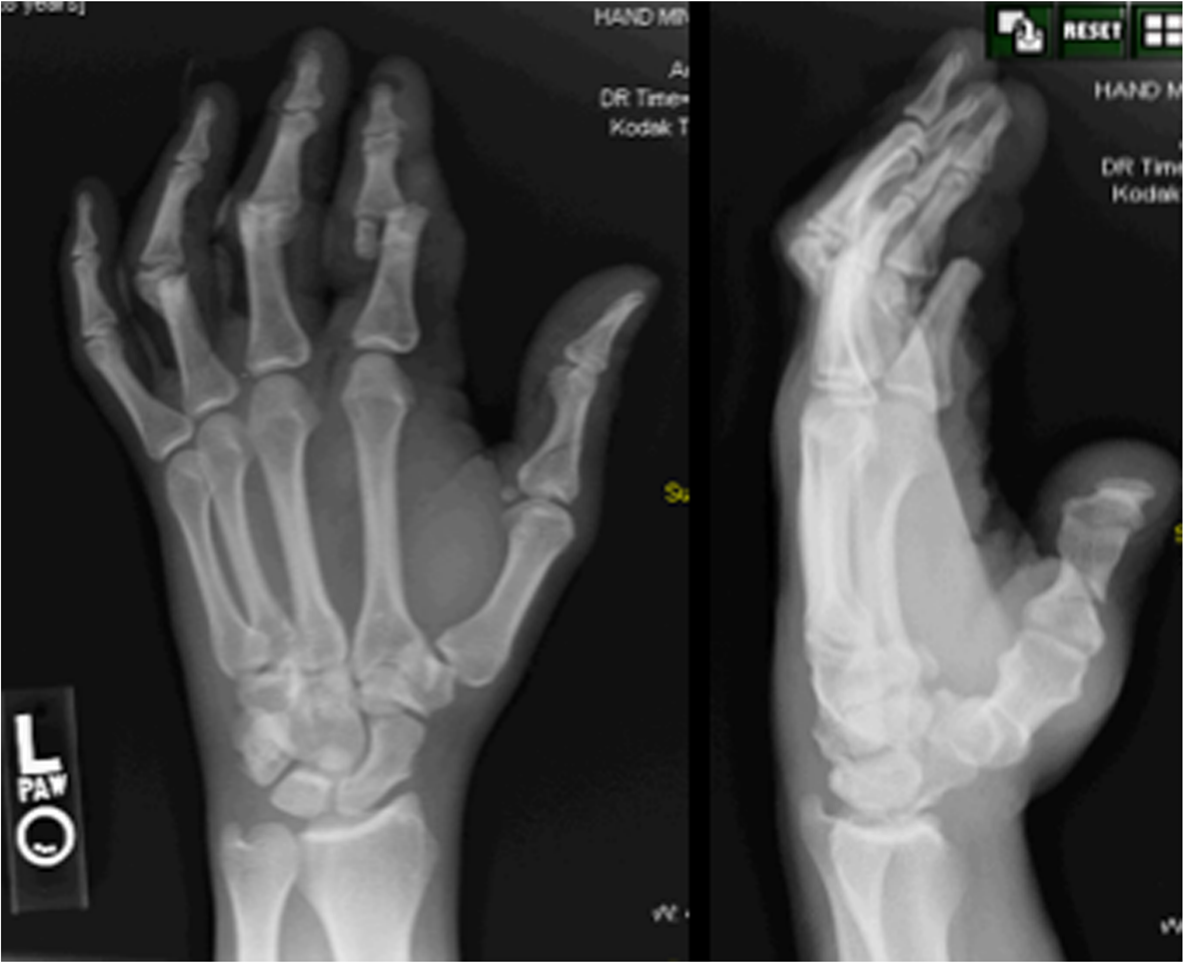
Preoperative radiographs showing proximal interphalangial joint fracture dislocations of left index, long, and ring fingers and proximal phalanx fracture of the thumb.

A 28-year-old right-hand-dominant man was airlifted for limb salvage to the level 1 trauma center where the investigators practice after sustaining an agricultural accident in which his left hand was pulled into a corn augur. The mangling injury of his hand included fractures of his index through ring fingers with extensive lacerations and exposed flexor tendons. There was a partial degloving injury to his left palm (Figures 1 and 2). The patient was otherwise stable and had no additional injuries. Upon arrival he was taken emergently to the operating room, where he underwent irrigation and debridement, primary fusion with Kirschner wires of the destroyed PIP joints in his left index, long, and ring fingers, a split-thickness skin-graft to his left palm and long finger from a left forearm donor site, and initiation of IV antibiotics, administering vancomycin and ampicillin/sulbactam. He recovered uneventfully and was discharged home four days later without additional antibiotic therapy.

**Figure 3:**
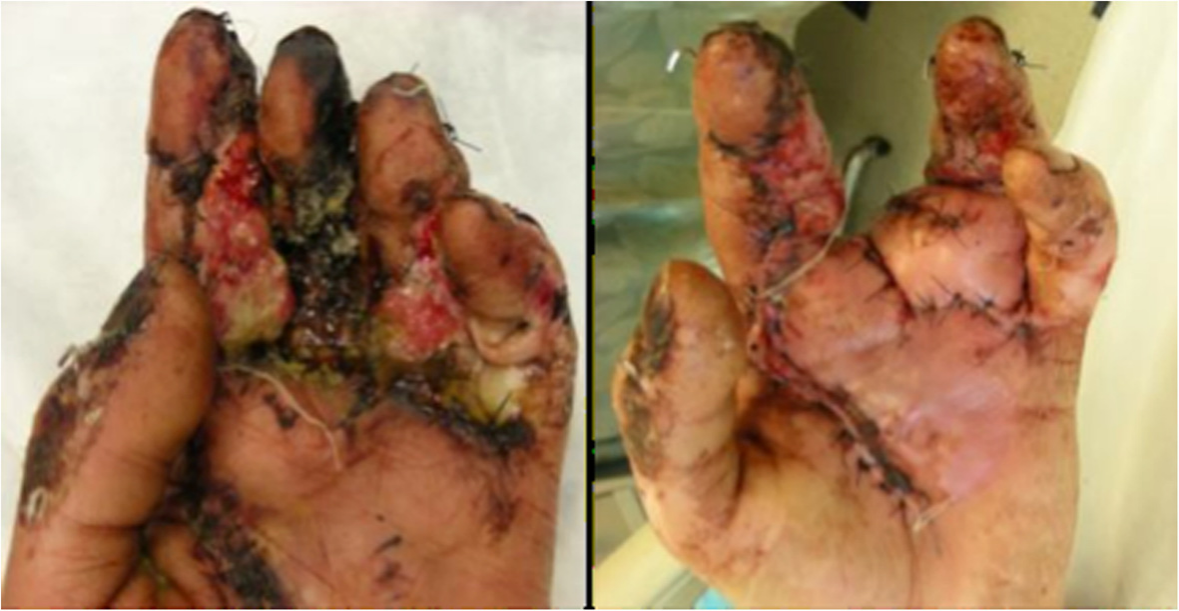
Day 14 showing full thickness necrosis of the volar aspect of the long finger and a deep infection at the volar base of the ring finger and distal palm (left). Postoperative aspect after long finger resection and filet flap coverage of volar defect with dorsal skin from long finger (right).

At his first follow-up appointment two weeks post-operatively, he showed no sign of systemic infection but was noted to have an extensive necrosis involving part of his left long finger and the palmar skin graft (Figure 3). Surgical debridement including amputation of the long finger was scheduled and he underwent a filet flap closure of the second webspace after long finger ablation. Tissue samples revealed both bacterial and mold growth, and the Infectious Disease team was consulted. Due to the suspicion for an invasive mucor mycosis he was taken the next morning for additional irrigation and debridement, and lipid-complex amphotericin B was added to his antibacterial medications. Cultures were speciated to coagulase-negative Staphylococcus, two different species of Pseudomonas, and mycotic species including Mucor and *Aspergillus fumigates*. He recovered well and was later discharged home on hospital day five with a Hohn catheter and an outpatient antibiotic regimen planned for four total weeks that included vancomycin 1 g intravenously twice daily, Zosyn 12 g continuous infusion daily, and Posaconazole suspension 400 mg (10 mL) orally twice daily.

He continued to show improvement at his follow-up appointments with both Orthopaedic Surgery and Infectious Disease. Approximately one week after discharge his AFB cultures taken intraoperatively turned positive by probe for *Mycobacterium tuberculosis* complex, which stood in contrast to a negative PPD test that had been documented within the previous two years. At this clinic visit a new PPD was placed and later found to be negative when read in his home town. A chest radiograph also showed no sign of TB infection. Ultimately the positive AFB was attributed to an environmental mycobacterial contaminant.

**Figure 4:**
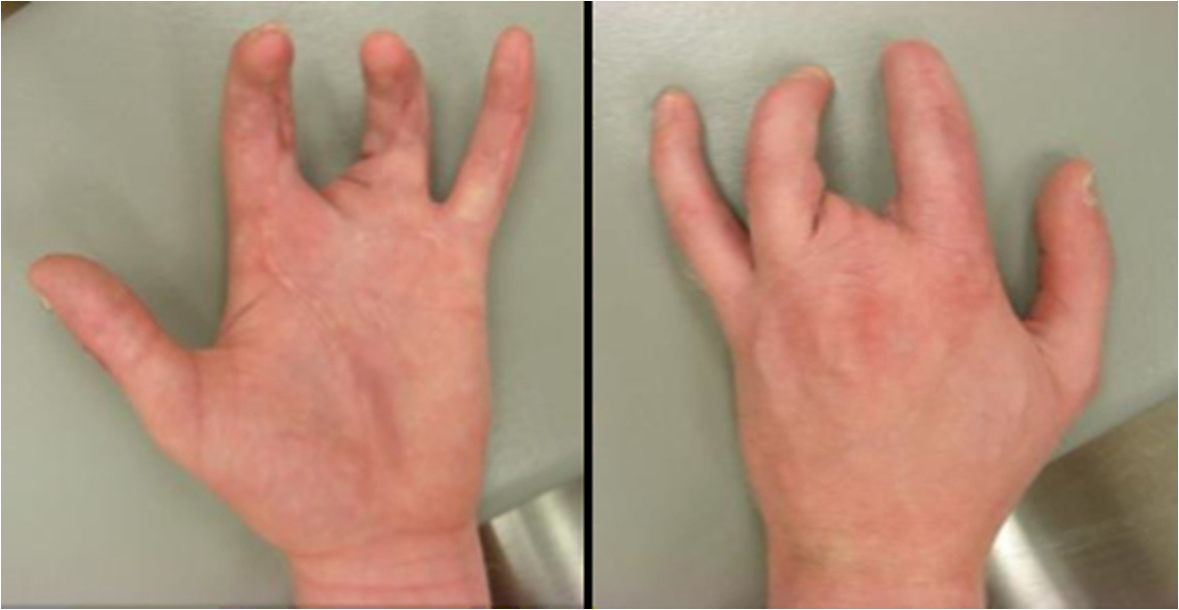
Follow up after 6 months showing healed soft tissues free of signs of infection.

His weekly labs drawn by a home health agency were otherwise unremarkable until approximately three weeks after his discharge. At that time he was found to be neutropenic with a white blood cell count of 3100 (absolute neutrophil count 120), and he was admitted to the hospital in his hometown where all of his antibiotics and antifungals were held. He was discharged several days later after recovery of his white blood cell count without sign of infection or any further complication. Though his antibiotic regimen was not restarted (as he had nearly completed his planned course), he remained free of infection and healed well (Figure 4).

## Conclusions

Mucormycosis is caused by organisms of the Mucorales order, which are commonly found in nature growing as mold on decaying plant matter and in the soil. The incidence of mucormycosis is not well-established, but it is estimated to be 1.7 cases per 1,000,000 people per year [2]. Given its ubiquity, the fact that the incidence of disease is so low is a testament to the effectiveness with which the normal immune system handles these organisms. Immunocompromised individuals more likely to be infected; conditions associated with increased risk of infection include poorly-controlled diabetes, hematopoietic malignancy, and solid organ/hematopoietic cell transplantation, among others [1]. There are infrequent case reports of mucormycosis following trauma [3-7].

Genera from the order Mucorales are responsible for most human infections caused by the fungal class Zygomycetes. Though these infections were previously termed either “mucormycosis” or “zygomycosis,” recent studies suggest that “mucormycosis” should be the correct name [8]. Human infections are most commonly attributed to Mucor, Rhizopus, Rhizomucor, though infections caused by other genera – including Absidia, Apophysomyces, Cunninghamella, and Saksenaea – have also been documented [1].

Mucorales organisms may cause infection via inhalation or ingestion of spores or by direct cutaneous inoculation. All demonstrate infarction and necrosis of infected tissue characterized by angioinvasion of fungal hyphae [9]. Mucormycosis most commonly affects the sinuses and lungs; skin is the third most common manifestation [10]. Our patient’s injury was the obvious source of his cutaneous inoculation, and the severity of the trauma itself likely contributed to his risk.

Cutaneous mucormycosis may present initially with varied appearances, including pustules/vesicles and widely necrotic wounds. Bouza *et al.* describe “a cotton-like growth that] may be observed over the surface of the tissues, a clinical sign known as ‘hairy pus [10].’” Our patient’s wounds did not demonstrate this sign, but were necrotic and purulent. Their appearance did not raise immediate suspicion for fungal infection specifically, but given the mechanism and setting of his injury it was important to consider fungal infection in his differential diagnosis, both on presentation and in the setting of post-operative infection.

While both medical and surgical measures are in the treatment of mucormycosis, prompt surgical debridement remains the mainstay. Obtaining tissue samples for histologic assessment is important in determining the likely pathogen: In our case the early return to the OR was indicated by the pathologic findings suspicious of murcormycosis. The European Society for Clinical Microbiology and Infectious Diseases (ESCMID) and the European Confederation of Medical Mycology (ECMM) recently published joint clinical guidelines for the diagnosis and management of mucormycosis, writing “surgery whenever possible is strongly recommended to be combined with medical treatment [11].”

Defining the extent of debridement necessary remains challenging, the capacity of mucormycosis to spread via angioinvasion may entail debridement so aggressive as to include amputation [12]. In our patient, amputation of the long finger and debridement to healthy-appearing bleeding tissue were sufficient to control the spread of infection; in more proximal lesions, this may be more challenging, as other authors have reported [13].

Standard antifungals may not be effective against mucormycosis. The ESCMID/ECCM paper recommends liposomal (or lipid-complex) amphotericin B as first-line antifungal therapy and recommends against the use of amphotericin B deoxycholate due to its side-effect profile. Posaconazole, a newer triazole agent, is recommended as salvage therapy for patients who fail primary antifungals due to refractory disease, intolerance, or both.

The basis for these recommendations is born out in previous case reports describing mucormycosis. Lineberry et al. recently reported success treating an upper extremity Rhizopus infection with surgical debridement in a 54-year-old woman without predisposing risk factors [4]. Seguin et al. recounted the case of a 39-year-old immunocompetent patient who required repeated debridements and ultimately above-knee amputation to control a lower leg wound infection with Absidia corymbifera [7]. Additionally, Moran et al. catalogued seven cases of upper extremity mucormycosis, four of which ultimately resulted in amputation; they concluded that early recognition and surgical debridement remains the central therapy in the treatment of these infections [3].

Even with surgery, these infections are so serious and difficult to manage that death may ultimately occur. Kontogiori et al. describe a fatal case of necrotizing fasciitis in a 25-year-old, previously healthy farmer who died 19 days after sustaining open lower extremity fractures infected by multiple organisms including Rhizopus despite once or twice daily debridement and aggressive antifungal therapy.6 Similarly Horre et al. reported the case of a 38-year-old otherwise healthy man who died 18 days after sustaining abdominal trauma in a bike accident that resulted in intraabdominal infection with Candida albicans and Absidia corymbifera; daily debridement and amphotericin B plus flucytosine failed to stop his infection.5.

In our patient, surgical debridement and lipid-complex amphotericin B were used while he was in-house, and posaconazole was substituted for amphotericin for his outpatient regimen given its oral route of administration (versus intravenous for amphotericin). No specific recommendations for duration of treatment were made in the ECSMID/ECMM report; our patient’s therapy was discontinued at four weeks after he became neutropenic. He remained free of any sign of infection at his follow-up six months later.

We attribute our success in managing this patient to prompt surgical management and appropriate antifungal therapy, and cannot overstate the importance of collaboration between our orthopaedic surgery and infectious disease teams in facilitating early recognition and treatment of this potentially devastating infection.

## Consent

Written informed consent was obtained from the patient for publication of this case report and any accompanying images. A copy of the written consent is available for review by the editor-in-chief of this journal. IRB approval was not required for this manuscript.

## Abbreviations

PIP joint: Proximal interphalangeal joint
IV: Intravenous
G: Gram
mL: milliliter
AFB cultures: Acid fast bacilli cultures
PPD test: Purified protein derivative test (Tuberculin test)
TB infection: Tuberculosis infection
ESCMID: European Society for Clinical Microbiology and Infectious Diseases
ECMM: European Confederation of Medical Mycology
